# The correlation study between blood urea nitrogen to serum albumin ratio and prognosis of patients with sepsis during hospitalization

**DOI:** 10.1186/s12871-022-01947-4

**Published:** 2022-12-28

**Authors:** Jie Min, Jianhong Lu, Lei Zhong, Meng Yuan, Yin Xu

**Affiliations:** 1 Department of Intensive Care Unit, Huzhou Central Hospital, Affiliated Central Hospital, Huzhou University, Huzhou, 313000 Zhejiang Province China; 2 Department of General Practice, Huzhou Central Hospital, Affiliated Central Hospital, Huzhou University, No.1558, North Sanhuan Road, Huzhou, 313000 Zhejiang Province China

**Keywords:** Blood urea nitrogen to albumin ratio, Sepsis, Intensive care unit, Prognosis, MIMIC-IV database

## Abstract

**Background:**

Sepsis is a common critical illness in intensive care unit (ICU) and seriously threatens the life of patients. Therefore, to identify a simple and effective clinical indicator to determine prognosis is essential for the management of sepsis patients. This study was mainly based on blood urea nitrogen to albumin ratio (B/A), a comprehensive index, to explore its correlation with the prognosis of sepsis patients during hospitalization.

**Methods:**

Totally, adult patients in ICU who were diagnosed with sepsis in Medical Information Mart for Intensive Care IV(MIMIC-IV) database from 2008 to 2019 were involved in this study. The study population were divided into survivors group and non-survivors group based on the prognosis during hospitalization. Restricted cubic spline (RCS) was utilized to analyze the association between B/A level and the risk of ICU all-cause mortality in patients with sepsis and determine the optimal cut-off value of B/A. The study population was divided into low B/A group and high B/A group based on the optimal cut-off value. The survival curve of ICU cumulative survival rate was draw through Kaplan–Meier method. The correlation between B/A and the prognosis of patients was conducted by multivariate Cox regression analysis. Furthermore, we performed sensitivity analyses to assess the robustness of the results.

**Results:**

A total of 10,578 patients with sepsis were enrolled, and the ICU all-cause mortality was 15.89%. The patients in the non-survivors group had higher B/A values and more comorbidities than those in the survivors group. RCS showed that the risk of ICU all-cause mortality increased with the B/A level, showing a non-linear trend (χ2 = 66.82, *p* < 0.001). The mortality rate in the high B/A group was significantly higher than that in the low B/A group (*p* < 0.001). Kaplan–Meier curves revealed that compared with the low B/A group, the ICU cumulative survival rate of patients with sepsis was significantly lower in the high B/A group (log-rank test, χ2 = 148.620, *p* < 0.001). Further analysis of multivariate Cox proportional hazards regression showed that an elevated B/A (≥ 7.93) was an independent factor associated with ICU mortality among patients with sepsis.

**Conclusions:**

An elevated B/A might be a useful prognostic indicator in patients with sepsis. This study could offer a deeper insight into treating sepsis.

**Supplementary Information:**

The online version contains supplementary material available at 10.1186/s12871-022-01947-4.

## Background

Sepsis is a common critical illness in intensive care unit (ICU) and is characterized by a dysregulated response to infection that leads to life-threatening organ dysfunction [1, 2]. It has been reported that about 1/3 of critically ill patients will develop sepsis [3]. While organ support and guidelines for sepsis management have improved, the rates of morbidity and mortality remain high, which have made sepsis a hot spot in critical care medicine. Therefore, identifying simple and effective clinical indicators to determine prognosis is essential for the management of sepsis patients.

Patients with sepsis generally suffer from elevated protein catabolism and are susceptible to acute kidney injury, which can result in elevated blood urea nitrogen (BUN).Studies have shown that elevated BNU can independently predict prognosis in critically ill patients [4, 5]. Albumin is the most abundant protein in human plasma, and patients with sepsis frequently have hypoalbuminemia. Lower albumin levels are interrelated to severe systemic inflammation and organ failure, resulting in poor outcomes [1, 6–8]. The ratio of blood urea nitrogen to serum albumin (B/A) is a new form of inflammation marker now being studied. It is calculated from the two indicators of urea nitrogen and albumin, which are easy to detect and inexpensive. Previous studies have reported that B/A has high clinical value in evaluating the prognosis of community-acquired pneumonia [9], acute pulmonary embolism [10] and chronic heart failure [11].

However, there is no research report on the correlation between B/A and the prognosis of patients with sepsis. This study was mainly based on B/A, a comprehensive index, to explore its correlation with the prognosis of patients with sepsis during ICU stay.

## Methods

### Data source

The study examined data from Medical Information Mart for Intensive Care IV (MIMIC-IV, v2.0) database, which is a large free database available to researchers worldwide. Institutional review boards at Massachusetts Institute of Technology (Cambridge, MA) and Beth Israel Deaconess Medical Center (Boston, MA) approved the creation of the database. Participants were all eligible adults with sepsis (2008 to 2019) in MIMIC-IV. The data of our study were extracted by one author (L Zhong), who completed a National Institutes of Health online training course and was authorized to access the data (certification number: 36142713).

### Study population

**Fig. 1:**
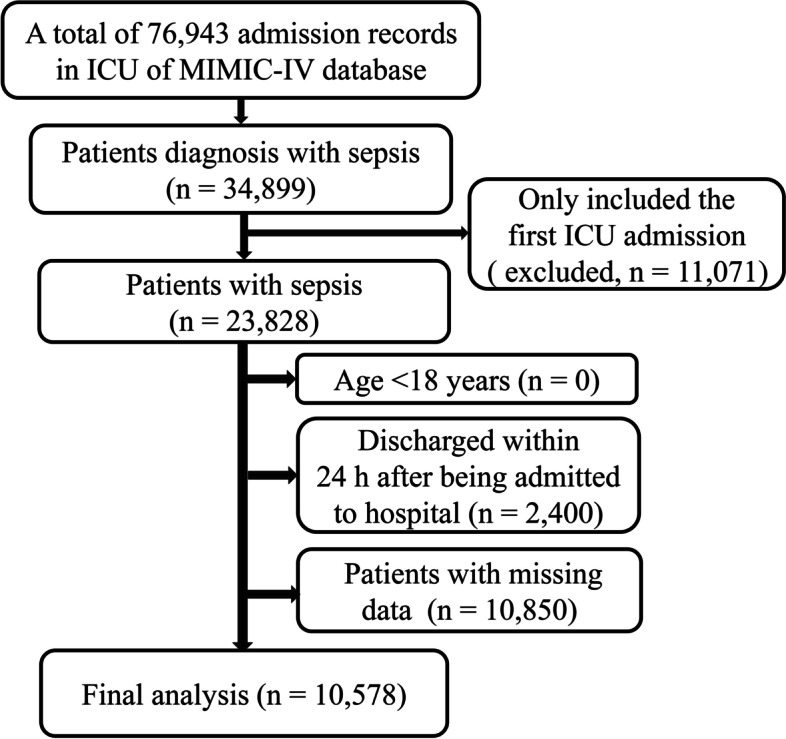
The screen process of study participants

In MIMIC-IV, there were 34,899 cases of sepsis (defined by Sepsis-3 criteria).In this study, patients (≥ 18 years) admitted to the ICU for the first time and diagnosed with sepsis were enrolled. Exclusion criteria were as follows: (1) ICU length of stay < 24 h; (2) Missing key data such as BUN and albumin. Sepsis 3.0 criteria defined sepsis as a suspected or confirmed infection with a sequential organ failure assessment (SOFA) score of 2 or higher [1], which was used for the diagnosis of sepsis in this study. A summary of the selection process for study participants was shown in Fig. 1.

### Data extraction

We used structured query language with PostgreSQL 10.13 to extract the MIMIC data. Data were collected as age, sex, SOFA score, comorbidities, length of hospital stay, and ICU mortality. In addition, laboratory parameters such as white blood cell (WBC), hemoglobin, platelet, red blood cell distribution width (RDW), glucose, creatinine, albumin, BUN, anion gap, K + , Ca2 + and Mg2 + were extracted. The B/A ratio (mg/g) was calculated based upon serum BUN (mg/dL)/serum albumin (g/dL). All laboratory data were extracted from the data collected within the first 24 h after the patient admitted to ICU. In the MIMIC IV database, the SOFA score was based on the data during the first 24 h after admission.

### Groups and outcomes

Patients were grouped into survivors (*n* = 8897) and non-survivors (*n* = 1681) based on the prognosis during hospitalization. In addition, we used restricted cubic splines (RCS) to determine the optimal cut-off value of B/A, and divided the study population into two groups: low B/A group (< 7.93, *n* = 5430) and high B/A group (≥ 7.93, *n* = 5148). ICU all-cause mortality was the study outcome measure.

### Statistical analysis

Continuous variables were presented as mean and standard deviation or median and interquartile ranges. Accordingly, Wilcoxon rank-sum test and T-test were used. The categorical variables were presented using percentages (%) and the Chi-squared test was applied to compare them.

RCS was utilized to analyze the association between B/A level at ICU admission and the risk of ICU all-cause mortality in patients with sepsis and determine the optimal cut-off value of B/A. The study population was divided into low B/A group and high B/A group based on the optimal cut-off value.

Kaplan–Meier curves were drawn, and the ICU cumulative survival rate was compared between the two groups using the log-rank test.

Variables with a *P*-value < 0.10 in univariate analysis were included in the multivariate Cox regression to analyze the prognostic value of B/A for clinical outcomes during ICU stay in patients with sepsis, and the results were presented as hazard ratio (HR) with 95% confidence interval (CI). Model I adjusted for nothing. Model II adjusted for age and SOFA score. Model III adjusted for age, SOFA score, anion gap, WBC, RDW, creatinine, glucose, K + , Mg2 + , cerebral infarction, chronic obstructive pulmonary disease (COPD), congestive heart failure (CHF), acute myocardial infarction (AMI), acute pancreatitis, hepatic failure and malignancy.

Stata14.0 software and R language were used for data analysis, and a two-sided *P*-value less than 0.05 was considered statistically significant.

### Sensitivity analysis

To assess the robustness of the results, we performed several sensitivity analyses. First, considering the infusion of human serum albumin before ICU admission, there may have an impact on the B/A value. Sensitivity analysis was performed after removing patients who had received human serum albumin infusion 3 days before ICU admission. Second, given the blunted inflammatory response to sepsis in the elderly, we performed another sensitivity analysis excluding patients younger than 60 years. Finally, we also performed a sensitivity analysis after removing patients with hepatic failure, considering the reduced hepatic synthetic capacity.

## Results

### Study population and baseline characteristics

A total of 10,578 patients with sepsis were enrolled, and the mean age was 64.70(16.79) years, 43.06% were female. Compared with survivors, non-survivors had higher age, SOFA score, blood urea nitrogen, B/A, anion gap, WBC, RDW, creatinine, blood glucose, K + and Mg2 + values. And COPD, CHF, AMI and hepatic failure were more common in non-survivors group. In contrast, the level of albumin, the incidence rate of acute pancreatitis and the length of hospital stay were higher in survivors group (all *p* < 0.05). A summary of the baseline characteristics for patients was shown in Table 1.

**Table 1:** A comparison of baseline characteristics between survivors and non-survivors

Variable	Overall population (*n* = 10,578)	Survivors (*n* = 8,897)	Non-survivors (*n* = 1,681)	t/Z/χ2 value	*P* value
Age (years)	64.70 ± 16.79	64.28 ± 16.89	66.89 ± 16.03	-5.854	< 0.001
Female [n (%)]	4,555 (43.06)	3,835 (43.10)	720 (42.83)	0.043	0.836
SOFA (score)	7.75 ± 4.08	7.22 ± 3.76	10.60 ± 4.48	-32.733	< 0.001
BUN (mg/dl)	22 (14, 38)	22 (14, 36)	28 (18, 47)	-12.394	< 0.001
Albumin (g/dL)	2.96 ± 0.64	2.99 ± 0.62	2.84 ± 0.70	8.771	< 0.001
B/A	7.69 (4.71, 13.93)	7.31 (4.58, 13.13)	10.29 (6, 18.33)	-14.170	< 0.001
Anion gap (mmol/L)	15.72 ± 4.85	15.35 ± 4.52	17.69 ± 5.93	-18.490	< 0.001
WBC (× 10^9^/L)	11.80 (8.00, 16.90)	11.70 (7.90,16.50)	13.40 (8.90, 19.00)	-8.482	< 0.001
Hemoglobin (g/L)	106.65 ± 23.43	106.61 ± 23.07	106.84 ± 25.25	-0.364	0.716
Platelet (× 10^9^/L)	181 (120, 254)	181 (122, 253)	181 (111, 258)	1.402	0.161
RDW (%)	15.43 ± 2.46	15.33 ± 2.39	15.96 ± 2.75	-9.667	< 0.001
Creatinine (umol/L)	97.24 (70.72, 159.12)	97.24 (70.72, 150.28)	114.92 (79.56, 194.48)	-11.624	< 0.001
Glucose (mmol/L)	7.33 (5.89, 9.67)	7.28 (5.89, 9.5)	7.83 (5.94, 10.67)	-4.674	< 0.001
K + (mmol/L)	4.22 ± 0.85	4.19 ± 0.83	4.36 ± 0.92	-7.460	< 0.001
Ca2 + (mmol/L)	2.03 ± 0.27	2.03 ± 0.25	2.03 ± 0.27	0.559	0.576
Mg2 + (mmol/L)	0.81 ± 0.20	0.80 ± 0.20	0.85 ± 0.22	-8.562	< 0.001
Comorbidities [n (%)]					
Hypertension	4,072 (38.49)	3,446 (38.73)	626 (37.24)	1.330	0.249
Diabetes	3,167 (29.94)	2,685 (30.18)	482 (28.67)	1.527	0.217
Cerebral infarction	1,255 (11.86)	1,034 (11.62)	221 (13.15)	3.145	0.076
COPD	1,446 (13.67)	1,161 (13.05)	285 (16.95)	18.268	< 0.001
CHF	2,829 (26.74)	2,343 (26.33)	486 (28.91)	4.791	0.029
AMI	865 (8.18)	668 (7.51)	197 (11.72)	33.391	< 0.001
Acute pancreatitis	292 (2.76)	262 (2.94)	30 (1.78)	7.090	0.008
Hepatic failure	712 (6.73)	463 (5.20)	249 (14.81)	207.929	< 0.001
malignancy	2,490 (23.54)	2,063 (23.19)	427 (25.40)	3.851	0.050
Length of hospital stay (days)	11.54 (6.58,19.88)	12.5(7.29,21.29)	6.67(2.88, 12.5)	27.184	< 0.001

*SOFA* sequential organ failure assessment, *BUN* blood urea nitrogen, *B/A* blood urea nitrogen to serum albumin ratio, *WBC* white blood cell, *RDW* red blood cell distribution width, *COPD* chronic obstructive pulmonary disease, *CHF* congestive heart failure, *AMI* acute myocardial infarction

### Correlation between B/A and ICU mortality

**Fig. 2:**
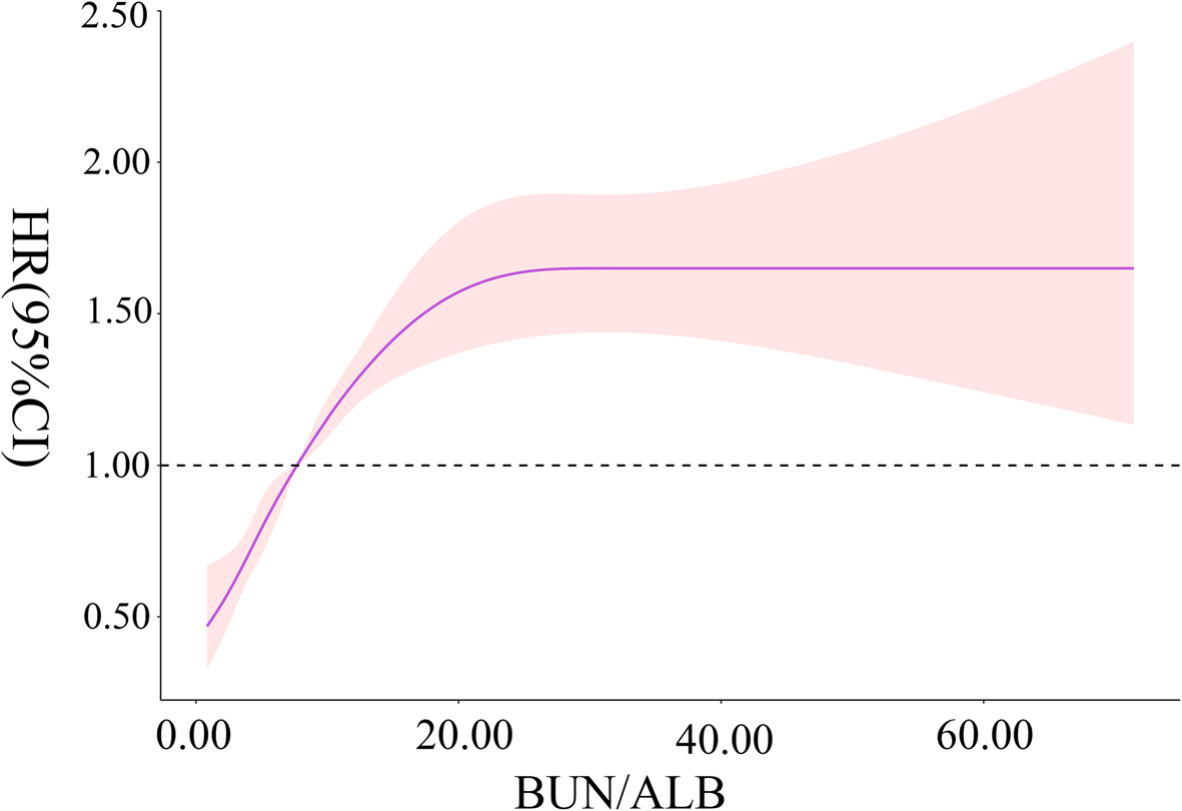
Association between BA level and the risk of ICU all-cause mortality in patients with sepsis

RCS showed that the B/A level at ICU admission had a non-linear trend with the risk of ICU all-cause mortality in patients with sepsis (χ2 = 66.82, *p* < 0.001). When the B/A level was 7.93, its HR was about 1. The risk of ICU all-cause mortality increased with the B/A level. When the B/A level was > 25.31, the risk of ICU all-cause mortality in patients with sepsis remained at a high level and was relatively stable, as shown in Fig. 2.

For the whole study population, the ICU all-cause mortality was 15.89%. We used RCS to determine the optimal cut-off value of B/A, and divided the study population into two groups: low B/A group (< 7.93, *n* = 5430) and high B/A group (≥ 7.93, *n* = 5148). The mortality rate in the high B/A group (20.38%) was significantly higher than that in the low B/A group (11.64%, χ2 = 150.951, *p* < 0.001), as indicated in Table 2.

**Table 2:** Comparison of ICU all-cause mortality between two groups

Group	Survivors (*n* = 8,897)	Non-survivors (*n* = 1,681)	χ2	*P*
Low B/A	4,798(88.36)	632(11.64)		
High B/A	4,099(79.62)	1,049(20.38)	150.951	< 0.001

*B/A* blood urea nitrogen to serum albumin ratio

**Fig. 3:**
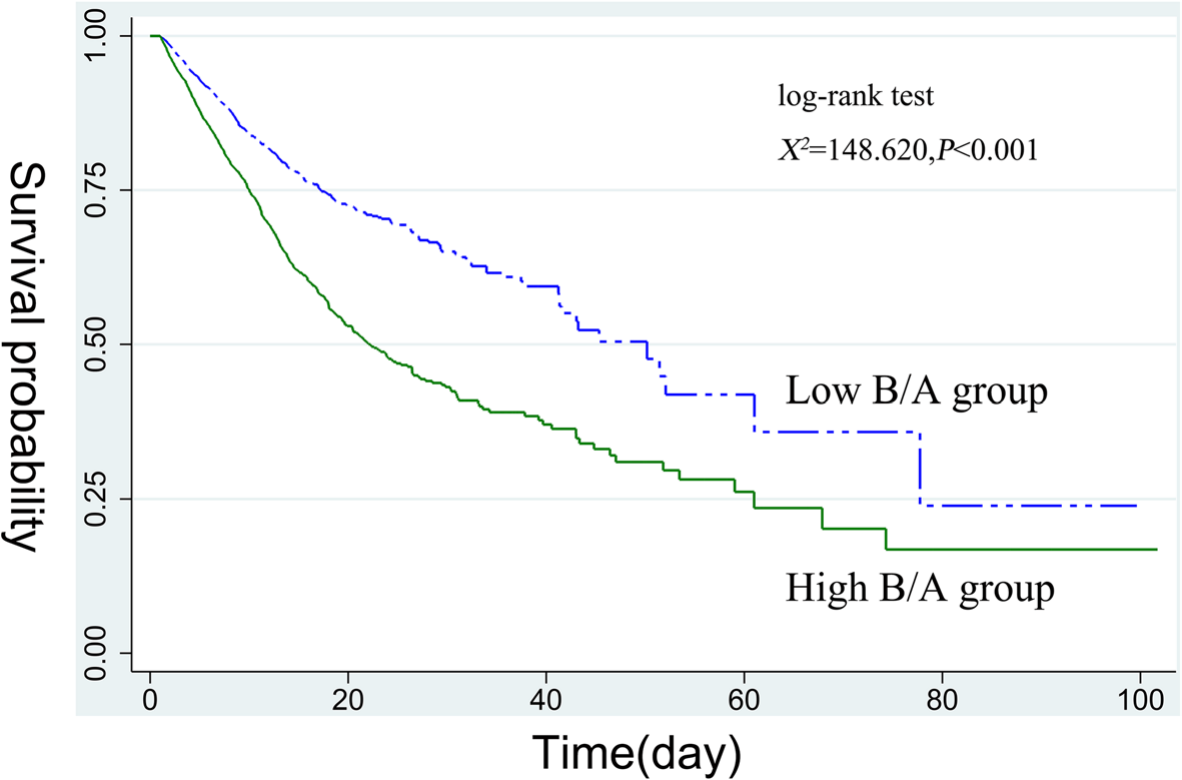
Kaplan–Meier curve of ICU cumulative survival rate for the low and high B/A groups. *B/A* blood urea nitrogen to serum albumin ratio

Next, we plotted the ICU survival curves for patients with sepsis based on the optimal cut-off value of B/A. Kaplan–Meier curves revealed that compared with the low B/A group, the ICU cumulative survival rate of patients with sepsis was significantly lower in the high B/A group (log-rank test, χ2 = 148.620, *p* < 0.001), as shown in Fig. 3.

Compared with the low B/A group, the HR (95% CI) of ICU all-cause mortality in the high B/A group was 1.832 (1.659–2.022), indicating that there was a significant association between an elevated B/A (≥ 7.93) and ICU all-cause mortality in patients with sepsis. This association remained statistically significant even after adjusting for age, SOFA score, anion gap, WBC, RDW, creatinine, glucose, K + , Mg2 + , cerebral infarction, COPD, CHF, AMI, acute pancreatitis, hepatic failure and malignancy, as shown in Table 3.

**Table 3:** Cox regression analysis of ICU all-cause mortality in patients with sepsis

Variable	Model I			Model II			Model III		
	**HR**	**95%CI**	***P***	**HR**	**95%CI**	***P***	**HR**	**95%CI**	***P***
**Low B/A**	1.0 (ref)			1.0 (ref)			1.0 (ref)		
**High B/A**	1.832	1.659–2.022	< 0.001	1.305	1.173–1.452	< 0.001	1.266	1.126–1.423	** < 0.001**

Model I adjusted for nothing

Model II adjusted for age and SOFA score

Model III adjusted for age, SOFA score, anion gap, WBC, RDW, creatinine, glucose, K + , Mg2 + , cerebral infarction, COPD, CHF, AMI, acute pancreatitis, hepatic failure and malignancy

*HR* hazard ratio, 95% *CI* 95% confidence interval, *B/A* blood urea nitrogen to serum albumin ratio, *SOFA* sequential organ failure assessment, *WBC* white blood cell, *RDW* red blood cell distribution width, *COPD* chronic obstructive pulmonary disease, *CHF* congestive heart failure, *AMI* acute myocardial infarction

### Sensitivity analysis

Three sensitivity analyses were performed to assess the robustness of our findings. After removing patients who had received human serum albumin infusion 3 days before ICU admission, sensitivity analyses showed that there was still a significant association between B/A and poor clinical outcomes in patients with sepsis (see Additional file 1). Furthermore, after excluding patients younger than 60 years, sensitivity analyses were performed again, and the results were consistent with our main finding (see Additional file 2). We then performed a sensitivity analysis after removal of patients with hepatic failure, and found that an elevated B/A (≥ 7.93) remained an independent factor associated with ICU mortality among patients with sepsis. An additional file showed this in more detail (see Additional file 3).

## Discussion

Sepsis is a syndrome of various pathogenic microorganisms invading the human body and causing systemic inflammatory response [12]. Early fluid resuscitation, infection control, and antibiotic therapy are the cornerstones of sepsis management aimed at correcting the condition of such patients [13]. Although sepsis guidelines have been continuously improved in recent years, and a series of active "rescue" measures have been taken internationally for sepsis, its morbidity and mortality remain high [14]. The mortality rate of sepsis patients hospitalized in ICU was as high as 41.9% [15]. Therefore, how to find an accurate and reliable clinical index, which is convenient for judging the prognosis of sepsis patients during hospitalization, so as to improve the successful rate of treatment, is still a major challenge for clinicians.

BUN is one of the end products of human protein catabolism and is mainly excreted in the kidney. It is an important marker representing renal function, metabolic status and nutritional status. In critically ill patients, protein catabolism is hyperactive and BNU synthesis increases during stress response. In addition, increased renal urea reabsorption leads to elevated blood urea nitrogen level in dehydrated states. In recent years, studies have reported its potential predictive value in poor clinical outcomes in patients with sepsis [16, 17]. In a retrospective cohort study of 12,713 patients with sepsis, there was a non-linear relationship between BUN and 30-day mortality [17]. Serum albumin, which is easily accessible as an indicator of nutritional status, contributes significantly to physiological homeostasis. Patients with sepsis commonly suffer from capillary leakage, reperfusion injury, tissue ischemia, and inflammatory responses, all of which can result in hypoalbuminemia [18, 19]. In patients with severe sepsis, lower albumin level has been linked with poor clinical outcomes and was an independent predictor of prognosis in patients with sepsis and septic shock, according to several studies [7, 20–22]. Our study showed that the non-survivors had a significantly higher BUN and lower albumin than those who survived during ICU hospitalization, which was in line with the other researches.

In recent years, blood urea nitrogen to albumin ratio (B/A) has been discovered to be an important prognostic biomarker, combining urea nitrogen with albumin, two important predictors. Previously, studies on the prognosis of disease based on B/A have focused primarily on lung diseases, such as pneumonia [9, 23, 24], pulmonary embolism [10], and lung cancer [25], and it has been established that elevated B/A had a high predictive value for mortality. The recent studies have also demonstrated that elevated B/A may play a role in prognosticating chronic heart failure [11], Escherichia coli bacteremia [26], and gastrointestinal bleeding [27], which also broaden our perspective. A study of 1253 elderly emergency department patients identified B/A as an independent predictor of ICU mortality. The comorbidities of the patients included chronic obstructive pulmonary disease, heart failure, and tumors. This also confirmed that B/A might have a certain predictive value for the prognosis of various diseases, and might even be an independent predictor of some severe diseases [28].

However, there is no research report on the B/A value for the prognosis evaluation of patients with sepsis, and this study may be the first report in this area. The results showed that the patients with sepsis in the non-survivors group had higher B/A values and more comorbidities than those in the survivors group. RCS showed that the risk of ICU all-cause mortality increased with the B/A level, showing a non-linear trend. The mortality rate in the high B/A group was significantly higher than that in the low B/A group. Kaplan–Meier curves revealed that compared with the low B/A group, the ICU cumulative survival rate of patients with sepsis was significantly lower in the high B/A group. Further analysis of multivariate Cox proportional hazards regression showed that an elevated B/A (≥ 7.93) was an independent factor associated with ICU mortality among patients with sepsis. In conclusion, this study provided a preliminary indication of correlation between elevated B/A levels and ICU all-cause mortality in patients with sepsis.

The study has several strengths compared to earlier works. First, this study, to our knowledge, might be the first report which conducted the value of B/A on the prognosis evaluation of patients with sepsis. Second, this study took the patients with sepsis in MIMIC-IV database as the research object, and was a large real-world study (10,578 sepsis patients). Based on the findings of this study, the prognosis of patients with sepsis can be evaluated clinically by monitoring the B/A level on admission. Early intervention in the process of clinical diagnosis and treatment can improve the prognosis of patients with sepsis, and finally we can achieve the goal of guiding clinical decision-making.

Although the sample size in this study was quite large, there were still some limitations. First, since the study was retrospective, selection bias and confounding bias were inevitable. Second, we merely calculated the initial B/A value of ICU patients after admission, and did not assess the changes in B/A value of patients during hospitalization. The levels of serum albumin or BUN may fluctuate over time, thus monitoring these values dynamically may be more accurate. In addition, the MIMIC database does not provide information regarding the etiology of sepsis or specific causes of death, which makes it impossible to study sepsis in a more comprehensive and detailed way. Finally, the results may be affected by confounding factors such as comorbidities. In order to verify if our findings were robust, we ran several sensitivity analyses. Even with these limitations, we found that B/A was associated with poor clinical outcomes in patients with sepsis. These findings were exploratory and therefore, a rigorously designed prospective study is necessary to evaluate and validate them.

## Conclusions

In conclusion, an elevated B/A (≥ 7.93) at ICU admission was an independent risk factor for poor prognosis in patients with sepsis, and it might have a certain application value in predicting death events of patients with sepsis during hospitalization. This study will be of help in early and effective evaluation of clinical outcomes in those patients and could offer a deeper insight into treating sepsis.

## Supplementary Information

**Additional file 1.** Sensitivity analysis after removing patients who had received human serum albumin infusion 3 days before ICU admission.

**Additional file 2.** Sensitivity analysis after excluding patients<60 years.

**Additional file 3.** Sensitivity analysis after removing patients with hepatic failure.

## Data Availability

The data that support the findings of this study are available from the Massachusetts Institute of Technology (MIT) and Beth Israel Deaconess Medical Center (BIDMC) but restrictions apply to the availability of these data, which were used under license for the current study, and so are not publicly available. Data are however available from the authors upon reasonable request and with permission of the Massachusetts Institute of Technology (MIT) and Beth Israel Deaconess Medical Center (BIDMC).

## Abbreviations

ICU: Intensive care unit
B/A: Blood urea nitrogen to serum albumin ratio
MIMIC-IV: Medical Information Mart for Intensive Care IV
RCS: Restricted cubic spline
BUN: Blood urea nitrogen
SOFA: Sequential organ failure assessment
WBC: White blood cell
RDW: Red blood cell distribution width
HR: Hazard ratio
CI: Confidence interval
COPD: Chronic obstructive pulmonary disease
CHF: Congestive heart failure
AMI: Acute myocardial infarction
