# Supplementary material for: The correlation study between blood urea nitrogen to serum albumin ratio and prognosis of patients with sepsis during hospitalization
Source: BMC Anesthesiol. 2022 Dec 28;22:404. doi: 10.1186/s12871-022-01947-4 (PMC9795581; doi:10.1186/s12871-022-01947-4)
Supplement: Supplementary file 2 — Additional file 2. Sensitivity analysis after excluding patients<60 years. [file 12871_2022_1947_MOESM2_ESM.docx]

**Additional file 2** Sensitivity analysis after excluding patients<60 years

| **Variable** | **model I** | | |  | **model II** | | |  | **model III** | | |
| --- | --- | --- | --- | --- | --- | --- | --- | --- | --- | --- | --- |
|  | **HR** | **95%CI** | ***P*** |  | **HR** | **95%CI** | ***P*** |  | **HR** | **95%CI** | ***P*** |
| **Low B/A (n=2,998)**  **High B/A (n=3,764)** | 1.0 (ref)  1.958 | 1.728 - 2.219 | <0.001 |  | 1.0 (ref)  1.556 | 1.365 - 1.774 | <0.001 |  | 1.0 (ref)  1.460 | 1.265 - 1.684 | <0.001 |

Model I adjusted for nothing.

Model II adjusted for SOFA score.

Model III adjusted for SOFA score, anion gap, WBC, RDW, creatinine, glucose, K+, Mg2+, cerebral infarction, COPD, CHF, AMI, acute pancreatitis, hepatic failure and malignancy.

*HR* hazard ratio, 95% *CI* 95% confidence interval, *B/A* blood urea nitrogen to serum albumin ratio, *SOFA* sequential organ failure assessment, *WBC* white blood cell, *RDW* red blood cell distribution width, *COPD* chronic obstructive pulmonary disease, *CHF* congestive heart failure, *AMI* acute myocardial infarction.
